# Prognostic significance of natural killer cell-associated markers in gastric cancer: quantitative analysis using multiplex immunohistochemistry

**DOI:** 10.1186/s12967-021-03203-8

**Published:** 2021-12-24

**Authors:** Hee Young Na, Yujun Park, Soo Kyung Nam, Jiwon Koh, Yoonjin Kwak, Sang-Hoon Ahn, Do Joong Park, Hyung-Ho Kim, Kyu Sang Lee, Hye Seung Lee

**Affiliations:** 1 Department of Pathology, Seoul National University Bundang Hospital, 173-82 Gumi-ro, Bundang-gu, Seongnam-si, Gyeonggi-do 13620 Republic of Korea; 2 Department of Pathology, Seoul National University College of Medicine, 103 Daehak-ro, Jongno-gu, Seoul, 03080 Republic of Korea; 3 Department of Pathology, Seoul National University Hospital, 101 Daehak-ro, Jongno-gu, Seoul, 03080 Republic of Korea; 4 Department of Surgery, Seoul National University Bundang Hospital, 173-82 Gumi-ro, Bundang-gu, Seongnam-si, Gyeonggi-do 13620 Republic of Korea; 5 Department of Surgery, Seoul National University College of Medicine, 103 Daehak-ro, Jongno-gu, Seoul, 03080 Republic of Korea

**Keywords:** Natural killer cell, NKG2A, Gastric cancer, Multiplex immunohistochemistry, Immunotherapy

## Abstract

**Background:**

Natural killer (NK) cells mediate the anti-tumoral immune response as an important component of innate immunity. The aim of this study was to investigate the prognostic significance and functional implication of NK cell-associated surface receptors in gastric cancer (GC) by using multiplex immunohistochemistry (mIHC).

**Methods:**

We performed an mIHC on tissue microarray slides, including 55 GC tissue samples. A total of 11 antibodies including CD57, NKG2A, CD16, HLA-E, CD3, CD20, CD45, CD68, CK, SMA, and ki-67 were used. CD45 + CD3-CD57 + cells were considered as CD57 + NK cells.

**Results:**

Among CD45 + immune cells, the proportion of CD57 + NK cell was the lowest (3.8%), whereas that of CD57 + and CD57- T cells (65.5%) was the highest, followed by macrophages (25.4%), and B cells (5.3%). CD57 + NK cells constituted 20% of CD45 + CD57 + immune cells while the remaining 80% were CD57 + T cells. The expression of HLA-E in tumor cells correlated with that in tumoral T cells, B cells, and macrophages, but not CD57 + NK cells. The higher density of tumoral CD57 + NK cells and tumoral CD57 + NKG2A + NK cells was associated with inferior survival.

**Conclusions:**

Although the number of CD57 + NK cells was lower than that of other immune cells, CD57 + NK cells and CD57 + NKG2A + NK cells were significantly associated with poor outcomes, suggesting that NK cell subsets play a critical role in GC progression. NK cells and their inhibitory receptor, NKG2A, may be potential targets in GC.

**Supplementary Information:**

The online version contains supplementary material available at 10.1186/s12967-021-03203-8.

## Background

Gastric cancer (GC) is one of the leading causes of cancer-related deaths worldwide [1]. The incidence rate varies geographically, which is high in East Asia (including Korea and China), Eastern Europe and South America and relatively lower in North America [2]. Although the survival rates of GC patients have increased with proper screening systems, standardized surgical protocols, and development of chemotherapy regimens, the general outcome of GC patients, especially those in advanced stages, remains poor. Therefore, numerous efforts have been made to find more effective treatments in GC.

The importance of the tumor immune microenvironment (TME) has been well-established in the last two decades [3]. Studies show that cancer cells undergo a series of sequential phases called the “elimination phase”, “equilibrium phase”, and “immune escape phase” [4]. In each phase, various immune cell populations and stromal cells in the TME and tumor cells play an important role by interacting with each other, leading to tumor suppression or progression [4]. Characterizing the interactions between immune cells and tumor cells mediated by diverse inhibiting or activating cell surface receptors and their ligands, especially in regard to their spatial relationship, is critical to understand the TME.

A recently developed multiplex immunohistochemistry (mIHC) technique, enables the comprehensive visualization of various cell populations with their spatial information, by analyzing high-throughput image data with computer software program, and is therefore a suitable tool for investigating the TME [5]. In contrast to mass cytometry (CyTOF) [6], and single-cell RNA sequencing [7], which are also reliable methods to elucidate the TME, mIHC is more practical and convenient to implement as it employs formalin fixed paraffin embedded block.

Among the various immune cells in the TME, cytotoxic T lymphocytes (CTLs) are one of the most important components which directly eliminate tumor cells [8]. Many researchers have demonstrated that a higher number of CD8 + CTLs is associated with better prognosis in various cancers, including colorectal cancer [9], and gastric cancer [10]. It is also well-established that upregulation of two critical inhibitory receptors of CTL, namely PD-1 and CTLA-4, leads to CTL exhaustion and thereby tumor progression [11]. Immune checkpoint inhibitors (ICIs) targeting the PD-1/PD-L1 axis or CTLA-4 can restore the anti-tumoral function of CTLs and impede tumor progression [12]. These drugs have been a breakthrough in many cancers, and the Food and Drug Administration (FDA) approved pembrolizumab (a PD-1 inhibitor) in locally advanced or metastatic gastric cancer with progression after 2 or more prior systemic chemotherapy [13]. Despite the safety and durable responses of these immunotherapeutic agents, only a subset of patients benefit from therapy [14], encouraging many researchers to explore new targets or alternative drug regimens by combining ICIs and other therapeutic agents.

Macrophages are also known as key modulators of tumor microenvironment. Macrophages can be differentiated into two extreme phenotype called M1 and M2 in response to the different stimuli [15]. M1 macrophages phagocytose and lyse tumor cells, and promote other immune cells by presenting tumor antigens [16], while M2 macrophages suppress anti-tumor immune response and contribute to tumor progression by promoting angiogenesis [17, 18]. The prognostic significance of macrophages in GC varies among studies which implemented conventional IHC method to detect surface expression of CD68 [19–22]. Compared to T cells and macropahges, the data regarding prognostic role of B cells in cancer are limited to date. In GC, some researchers demonstrated positive prognostic significance [23, 24] while others reported no association with prognosis [19].

Natural killer (NK) cells, that eliminate infected cells or tumor cells as a component of innate immunity [25], along with their various cell surface ligands, have recently emerged as a potential therapeutic target in solid tumors in the way of exploring novel targets in immunotherapy. Among different NK cell-associated surface markers, CD57 is expressed in mature NK cells that are less proliferative but more actively cytotoxic [26]. Besides these surface markers, NK cells also express various activating or inhibitory receptors, such as CD16 and NKG2A. CD16 is one of the most potent activating receptors of NK cells, which recognize tumor antigens and kill the tumor cells [27]. In contrast, NKG2A is an inhibitory receptor that recognizes HLA-E molecules on the tumor cells and lead to immune tolerance [28]. The prognostic significance of NK cells has been robustly studied by many researchers. Although higher NK cell density has been described as an indicator of good prognosis in multiple cancers, including colorectal cancer [29], and GC [30], contradictory results have been reported in breast cancer [31], and glioblastoma [32]. This discrepancy might result from the use of different antibodies such as CD56, CD57, or NKp46, which are used to detect the NK cell population. Moreover, all previous studies used conventional IHC methods, which made it difficult to analyze the subpopulation of NK cells expression specific receptors.

In the present study, we aimed to evaluate the prognostic significance of various immune cells or subset of immune cells with special regard to the NK cell-associated surface markers and receptors. We investigated the proportion of CD57 + NK cells (CD45 + CD3- CD20- CD57 + CD16 ± /NKG2A ±), T cells (CD45 + CD3 + CD20- CD57 ±), B cells (CD45 + CD3- CD20 +), and macrophages (CD45 + CD3- CD20- CD57- CD68 +) in GC by comprehensive visualization and analysis using mIHC.

## Methods

### Case selection

A total of 55 cases of consecutive stage II-III GC surgically resected between 2006 and 2008 at Seoul National University Bundang Hospital (SNUBH) were recruited for the study. The FFPE tissue samples were reviewed, and representative tissue areas were dissected for the construct a tissue microarray (TMA). Clinicopathological information was retrieved from electronic medical records.

### Reagents and resources

All reagents, staining devices and software for the image analyses used in this study are listed in Additional file 1: Table S1. The antibodies used in this study and the staining order are also listed in Additional file 2: Table S2.

### mIHC and computer-assisted image analyses

All implementations and analysis of mIHC were performed on the SuperBioChips (SuperBioChips Laboratories) as previously described [5]. After obtaining the digitally scanned images, the results of mIHC were analyzed, using publicly available software list in Additional file 2: Table S2. To quantify the amount of tumor-infiltrating immune cells, the cut-off values for each antibody were designated by either manual inspection of images or by analyzing the distribution of staining intensity of cells. Immune cells in the CK-positive tumor cell area were considered tumoral immune cells, whereas immune cells that are not in the CK-positive tumor cell area were considered stromal immune cells. Cell type of immune cells was determined based on lineage-specific antibody expression (Table 1). After quantification, the numbers of each cell type from different GC cases were compared and correlations with clinicopathological characteristics were investigated.

**Table 1:** Determination of immune cell types

Cell type	IHC profile
T cell	CD45 + CD3 + CD20- (CD57 + or CD57-)
B cell	CD45 + CD3- CD20 + CD57-
CD57 + NK cell	CD45 + CD3- CD20- CD57 +
Activating ligand	CD16
Inhibiting ligand	NKG2A
Macrophage	CD45 + CD3- CD20- CD57- CD68 +

*IHC* immunohistochemistry, *NK* natural killer

### Conventional IHC and EBV in situ hybridization (ISH)

For molecular classification of 55 GC samples, conventional immunohistochemistry (IHC) for E-cadherin (clone 36, mouse monoclonal; BD Biosciences, San Jose, CA, USA) and p53 (DO7, mouse monoclonal; Dako;Agilent Technologies, Santa Clara, CA) was performed on 3-μm-thick TMA slides using an automated immunostainer (BenchMark XT; Ventana Medical Systems) according to the manufacturer’s protocol. EBV ISH was performed using the INFORM EBV-encoded RNA probe (Ventana Medical Systems). The results of conventional IHC and EBV ISH were interpreted by two pathologists (J.K. and H.S.L.) in blinded manner. Discrepant cases were discussed to reach a consensus. For E-cadherin, strong membranous staining in tumor cells was defined as positive expression, whereas complete loss of membranous staining or aberrant cytoplasmic staining was interpreted as altered expression. For p53, strong nuclear staining in more than 10% of the tumor cells was defined as p53 positive. Cases were considered p53 negative when less than 10% of tumor cells were positive, and samples showing weak, variably scattered, or patchy positive tumor cells were determined as negative [33].

### Microsatellite instability (MSI) test

The MSI test was performed according to the revised Bethesda guidelines [34]. Polymerase chain reaction (PCR) amplification of the extracted DNA from tumor and normal cells was performed and the PCR products were analyzed using a DNA autosequencer (ABI 3731 Genetic Analyzer, Applied Biosystems, Foster City, CA). Allele profiles of five markers (BAT-26, BAT-25, D5S346, D17S250, and S2S123) in tumor cells were compared to those of matched normal cells. Tumors with additional alleles in two or more markers were classified as MSI-high (MSI-H), tumors with novel bands in one marker were defined as MSI-low (MSI-L), and those with identical bands in all five markers were classified as microsatellite stable (MSS).

### Molecular classification of GCs

We classified 55 GCs into 5 subtypes according to conventional IHC, EBV ISH, and MSI status as previously described [35]. A total of 4, 7, 21, 4, and 19 cases were classified as EBV + , MSI-H, EBV-MSS epithelial-mesenchymal transition (EMT)-like, EBV- MSS non-EMT-like p53 + , and EBV- MSS non-EMT-like p53- type, respectively.

### Statistical analyses

All analyses were performed using R 4.0.3, RStudio 1.3.1093, and SPSS 25.0 (SPSS, Inc., Chicago, IL, USA). For mIHC, the t-test was used to identify statistical significance between immune cells, molecular classification, and clinical information. Spearman's rank correlation was used to analyze the correlation between immune cell density and human leukocyte antigen (HLA)-E expression in tumor cells. Linear regression analysis was performed using the ‘lm() function of the “stats” package. For univariate survival analysis of immune cell context, the ‘coxph()’ function of the package “survival” (Terry M Therneau (2021), R package version 3.2–10; https://cran.r-project.org/web/packages/survival/index.html) was used. The ‘surv_cutpoint()’ function of the package “survminer” (Alboukadel Kassambara (2021), R package version 0.4.9; https://cran.r-project.org/web/packages/survminer/index.html) was used to identify the ideal cut-off for determining the high and low levels for each immune cells [36]. A probability value of less than 0.05 was considered statistically significant.

## Results

### Patient characteristics

The clinicopathological characteristics are summarized in Table 2. There were 31 (56.4%) male and 24 (43.6%) female patients with a median age of 55 years (range, 30–70 years). Histologic diagnoses showed tubular adenocarcinoma in 50 (90.9%), mucinous adenocarcinoma in 4 (7.3%), and poorly cohesive carcinoma (signet ring cell carcinoma) in 1 (1.8%) of the cases. A total of 14 (25.5%) cases were stage II and 41 (74.5%) cases were stage III. Median progression-free survival (PFS) and overall survival (OS) were 65.4 months (range, 4.0–102.9 months) and 85.4 months (range, 13.3–107.9 months), respectively.

**Table 2:** Clinicopathological characteristics of 55 patients

Characteristics	N = 55
Age (median, range) (years)	55 (30–77)
Gender	
Male	31 (56.4%)
Female	24 (43.6%)
Diagnosis (WHO 2010)	
Tubular adenocarcinoma	50 (90.9%)
Mucinous adenocarcinoma	4 (7.3%)
Poorly cohesive carcinoma (Signet ring cell carcinoma)	1 (1.8%)
WHO grade	
Well differentiated	0 (0.0%)
Moderated differentiated	20 (36.4%)
Poorly differentiated	34 (61.8%)
Undifferentiated	1 (1.8%)
Lauren classification	
Intestinal	19 (34.5%)
Diffuse	36 (65.5%)
Lymphatic invasion	43 (78.2%)
Perineural invasion	46 (83.6%)
Venous invasion	15 (27.3%)
Microsatellite instability high	6 (10.9%)
EBV infection	4 (7.3%)
Stage (AJCC 7th)	
II	14 (25.5%)
III	41 (74.5%)
Disease progression	23 (41.8%)
Death	21 (38.2%)
PFS (months) (median, range)	65.4 (4.0–102.9)
OS (months) (median, range)	85.4 (13.3–107.9)

*WHO* world health organization, *AJCC* American joint committee of cancer, *PFS* progression-free survival, *OS* overall survival

### Immune cell composition in gastric carcinoma focusing on NK cells and NK cell receptors

**Fig. 1:**
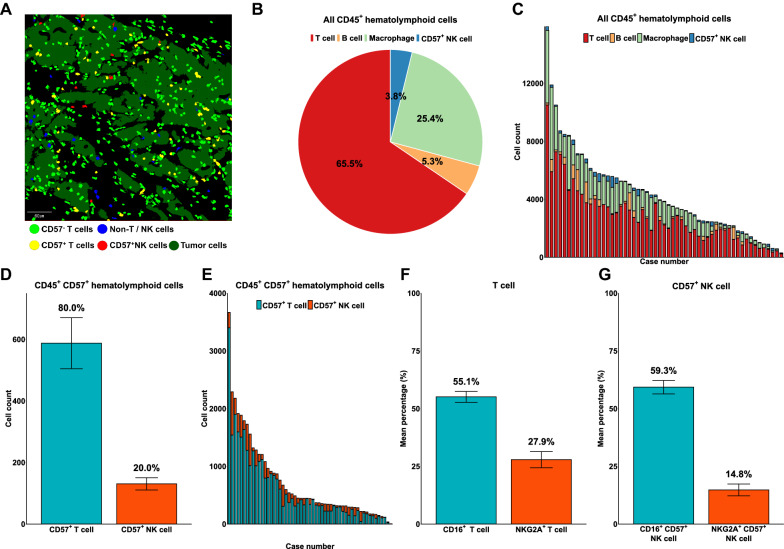
Immune cell composition in gastric cancer with an emphasis on NK cell-associated markers. CD57- T cells, CD57 + T cells, CD57 + NK cell, non-T/NK immune cells are presented as green, yellow, red and blue color (**A**). Tumor and stromal area were marked dark green and black color, respectively. The proportion of T cells, B cells, macrophages, and CD57 + NK cells is as depicted (**B**). The proportion of each immune cells in each case is illustrated (**C**). Among CD45 + CD57 + immune cells, CD57 + T cells and CD57 + NK cells constituted 80% and 20%, respectively (**D**). The proportion of CD57 + T cells and CD57 + NK cell among CD45 + CD57 + immune cells in each case is illustrated (**E**). A total of 55.1% and 27.9% of T cells showed CD16 and NKG2A expression (**F**). A total of 59.3% and 14.8% of CD57 + NK cells expressed CD16 and NKG2A (**G**)

We performed chromogenic mIHC in 55 stage II-III GCs to investigate the immune context focused on NK cell-associated surface markers in GC. The densities of CD57 + T cells (yellow), CD57- T cells (green) and CD57 + NK cells (red) were determined in each case (Fig. 1A and Additional file 3: Figure S1). Among CD45 + immune cells, the proportion of CD57 + NK cells was the lowest (3.8%), while the proportion of T cells (65.5%) was the highest, followed by macrophages (25.4%) and B cells (5.3%) (Fig. 1B). The median densities of T cells, B cells, CD57 + NK cells and macrophages were 1281.1/mm^2^, 38.7/mm^2^, 44.6/mm^2^, and 469.1/mm^2^, respectively (Additional file 4: Table S3). The proportions of T cells, B cells, macrophages, and CD57 + NK cells varied in each case (Fig. 1C). Among CD45 + CD57 + immune cells, 80.0 ± 2.1% also expressed CD3, leaving only 20.0 ± 2.1% CD57 + NK cells (Fig. 1D). The proportion of CD57 + T cells and CD57 + NK cells among CD45 + CD57 + immune cells also varied in each case (Fig. 1E).

Regarding the NK cell-associated activating and inhibitory receptors CD16 and NKG2A, 55.1 ± 2.34% of T cells were expressing CD16, while 27.9 ± 3.5% showed NKG2A expression (Fig. 1F). Similarly, 59.3 ± 2.9% of CD57 + NK cells were positive for CD16, and 14.8 ± 2.6% were positive for NKG2A (Fig. 1G). Of note, CD57 + NK cells constituted 2.4 ± 0.3% and 3.8 ± 0.8% of CD16 and NKG2A expressing immune cells (Additional file 5: Figure S2A and S2B). T cells constituted majority of CD16 and NKG2A expressing immune cells, followed by macrophages, CD57 + NK cells and B cells (Additional file 5: Figure S2A and S2B). The detailed numbers of immune cells are listed in Additional file 4: Table S3.

### Correlation with molecular classification and other clinicopathological characteristics

**Fig. 2:**
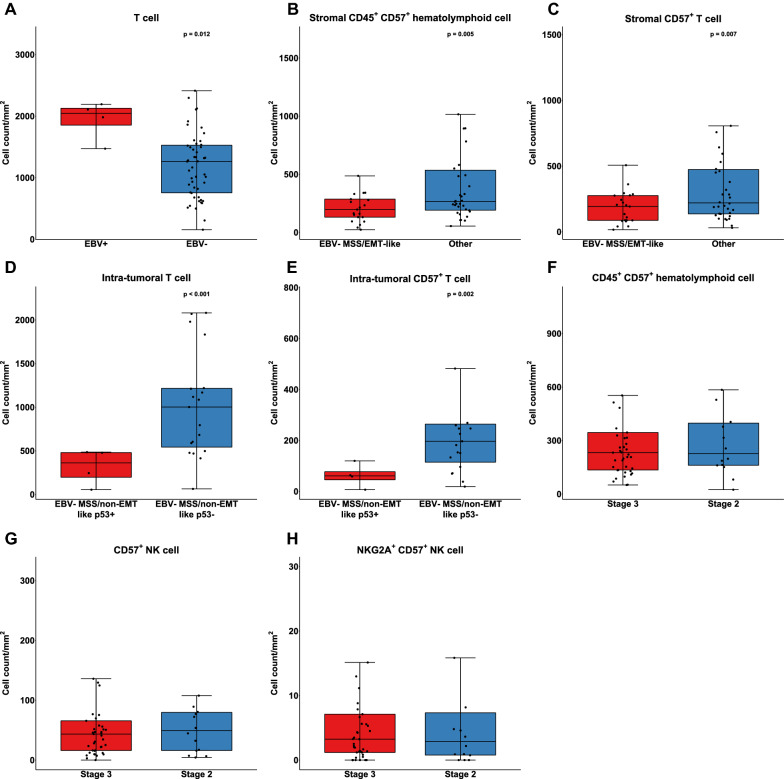
Correlation with molecular classification and other clinicopathological characteristics. T cell density was higher in EBV + gastric cancer than in EBV- gastric cancer (**A**). CD57 + immune cell (**B**) and CD57 + T cell (**C**) density was lower in EBV- MSS/EMT-like gastric cancer than in other molecular subtypes. T cell density (**D**) and CD57 + T cell density (**E**) was higher in EMV- MSS/non-EMT-like p53- subtype than in EBV- MSS/non-EMT-like p53 + subtype. CD45 + CD57 + immune cell (**F**), CD57 + NK cell (**G**), and CD57 + NKG2A + NK cell (**H**) density did not differ between stage 3 and stage 2 gastric cancer

When compared with EBV- GCs, EBV + GCs harbored a significantly higher number of T cells (p = 0.012) (Fig. 2A). There was no significant difference in the number of other immune cells between the two groups (Additional file 6: Figure S3A – S3C). The number of T cells, CD57 + NK cells and macrophages was higher in MSI-H GCs than in MSS/MSI-L GCs although the differences were not statistically significant (all p > 0.05) (Additional file 6: Figure S3D – S3F). When immune cells were segregated based on location, the density of stromal CD57 + immune cells and CD57 + T cells was significantly lower in EBV- MSS EMT-like GCs than in other 4 subtypes (Fig. 2B and 2C). And the number of tumoral T cells and tumoral CD57 + T cells was higher in EBV- MSS non-EMT-like p53- GCs than in EBV- MSS non-EMT-like p53 + counterpart (Fig. 2D and 2E).

The density of CD57 + immune cells, CD57 + NK cells and CD57 + NKG2A + NK cells did not correlate with other clinicopathological characteristics, including stages (Fig. 2F–H), lymphovascular invasion and degree of tumor differentiation.

### Correlation among immune cell densities and HLA-E expression in tumor cells

**Fig. 3:**
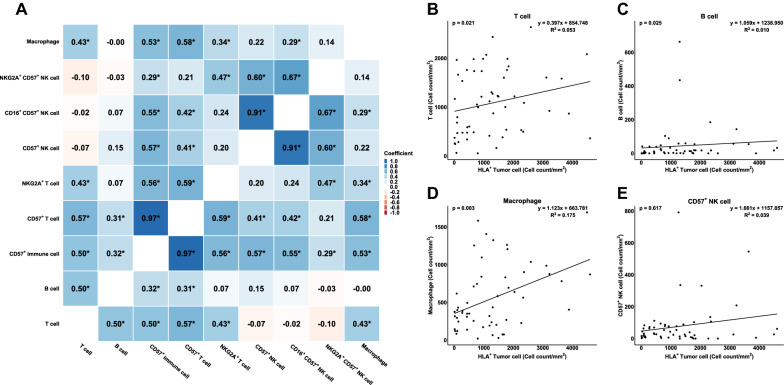
A correlation between immune cells are illustrated in heatmap (*: P < 0.05 by Spearman's Rank-Order Correlation) (**A**). HLA-E + tumor cells correlated with T cells (**B**), B cells (**C**), macrophages (**D**), and CD57 + NK cells (**E**)

The density of T cells correlated with that of B cells, and macrophages (all p < 0.05). Notably, the density of CD57 + NK cells showed correlation with the density of CD57 + lymphocytes and CD57 + T cells (all p < 0.05) (Fig. 3A) and not with T cells, B cells, and macrophages (all p > 0.05).

We also analyzed whether HLA-E expression in tumor cells displayed any correlation with immune cell density. The median number of tumor cells with HLA-E expression per mm^2^ was 1085.3 (range 10.7 – 4602.1). The expression of HLA-E correlated with the densities of intratumoral T cells, B cells, and macrophages (all p < 0.05), whereas it did not correlate with CD57 + NK cell density or CD57 + NKG2A + NK cell density (all p > 0.05) (Fig. 3B–E).

### Survival analysis

**Fig. 4:**
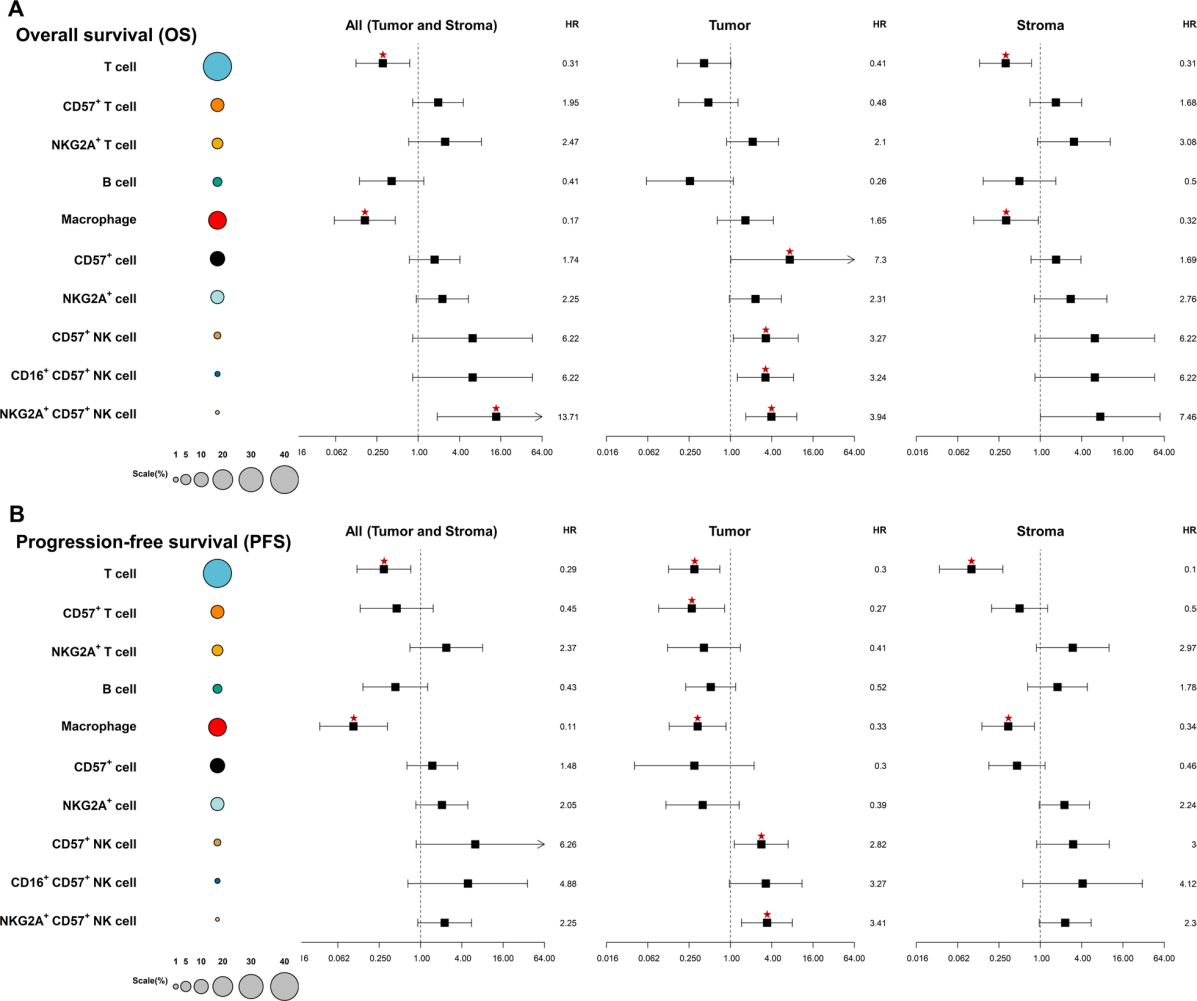
A univariate survival analysis representing hazard ratio of each immune cell type or a subset of immune cells. The proportion of each immune cell types or a subset of immune cells in all immune cells were represented with the size of circle. Tumoral CD57 + NK cells were associated with inferior overall survival (**A**) and progressive-free survival (**B**)

Univariate Cox regression analysis was performed to find out which immune cells or subset of immune cells are associated with prognosis. For each immune cells, the significance of the density of tumoral and stromal immune cells was investigated. In addition, the significance of the density of total (“tumoral and stromal”) immune cells was also analyzed. Higher density of “tumoral and stromal” and stromal T cells and macrophages was associated with longer OS (all p < 0.05) (Fig. 4A). High tumoral CD57 + NK cells, CD16 + CD57 + NK cells, and NGS2A + CD57 + NK cells was associated with shorter OS (all p < 0.05) (Fig. 4A). Likewise, high “tumoral and stromal”, tumoral, and stromal T cells and macrophages was associated with longer PFS (all p < 0.05) (Fig. 4B). Tumoral CD57 + T cells was associated with longer PFS (p < 0.05). And higher density of tumoral CD57 + NK cells, and NKG2A + CD57 + NK cells was associated with superior PFS (all p < 0.05) (Fig. 4B). High density of B cells showed a tendency of longer OS and PFS, but the differences were not statistically significant (Fig. 4A, B).

## Discussion

In the current study, we uncovered the TME of GCs by using mIHC, with a special reference to NK cells and their surface receptors. Comprehensive analysis of TME revealed that high density of T cells and macrophages are associated with better prognosis, similar with previous studies. Although NK cells constitute a minor component of tumor-infiltrating immune cells, the density of tumoral CD57 + NK cells and CD57 + NKG2A + NK cells was significantly associated with inferior OS and PFS in our cohort. This suggests that CD57 + NK cells in GC are not actively cytotoxic, which might be related to the expression of NKG2A or possibly other inhibitory receptors.

ICIs targeting PD-1/PD-L1 [37] and CTLA-4 [38] has proved to be a breakthrough in cancer treatment in the last decade. However, only a minority of patients benefit from these agents, thereby necessitating patient selection and new target discovery [39]. NK cells and their surface receptors have been proposed as novel immunotherapeutic targets [40]. Although the prognostic significance of NK cells in various cancers has been studied, the results are discrepant and NK cell receptors CD16 or NKG2A have not been thoroughly investigated. Moreover, most previous studies used conventional IHC, which made detailed subgrouping of immune cells according to their receptor expression impossible [29–32, 41, 42]. Therefore, we aimed to thoroughly investigate the expression of activating or inhibitory receptors in NK cells and their prognostic significance in GC.

NK cells are key to innate immunity and can kill cancer cells as well as infected cells faster than T cells [27]. NK cells are characterized by the expression of CD56 and lack of CD3 expression and can be subdivided into less mature CD56^bright^ and more mature CD56^dim^ subsets [43]. CD57, which is induced in the CD56^dim^CD16 + subset, is a marker of terminal differentiation [44]. CD57 + NK cells are less proliferative but more actively cytotoxic. Among various surface receptors, CD16 is one of the most potent activating receptors of NK cells, which recognize tumor antigens via immunoglobulin G and lead to antibody-dependent cell-mediated cytotoxicity [27]. In contrast, NKG2A is an inhibitory receptor that recognizes HLA-E molecules and prevents NK cell activation [28].

By using mIHC, including anti-CD57, anti-CD16, anti-NKG2A antibodies, and other immune cell markers, we observed that CD57 + NK cells constituted only 3.8% of all immune cells in GC. Of interest, 80% of CD45 + CD57 + immune cells were T cells, leaving only 20% as CD57 + NK cells. In addition, CD57 + NK cells are a minor component of CD16 + or NKG2A + immune cells. Our results confirmed that the number of CD57 + NK cells is far lower than that of T cells in GC [45]. It is also well known that CD57 [46] and NKG2A [47] are expressed CD8 + T cells as well as NK cells. Chen et al. showed that CD8 + T cells predominate NKG2A + lymphocytes in lung cancer [48].

In univariate Cox regression analysis, a higher density of T cells and macrophages was associated with longer OS and PFS. These results are similar to previous analyses, which demonstrated a high number of T cells as an indicator of favorable prognosis in GC patients [49–51]. Interestingly, “tumoral and stromal”, and “stromal” CD57 + T cells were associated with inferior OS, although it was not statistically significant, while tumoral CD57 + T cells were associated with longer PFS. Although the majority of CD57 + T cells are CD8 + cytotoxic T cells, the cytotoxic activity of CD8 + CD57 + T cells depend on the expression of CD27 and CD28. CD8 + CD57 + T cells in peripheral blood which do not express CD27 and CD28 have enhanced cytotoxic potential, while CD27 and CD28 expressing CD8 + CD57 + T cells in tumor have impaired cytotoxic activity [52]. Although we did not investigate the expression of CD27 and CD28 in tumoral or stromal CD57 + T cells, differential expression of CD27 and CD28 in tumoral and stromal CD57 + T cells might be one possible explanation for our results. Regarding macrophages, the prognostic significance varies greatly among studies in which some researchers reported them as good prognostic factor [19, 20] while others demonstrated contradicting results [21, 22] by using single anti-CD68 antibody. When macrophages were subdivided into M1 and M2 subtypes, M1 macrophages were generally associated with good prognosis [53, 54] whereas M2 subtypes were enriched in cases showing poor outcomes [55–58]. In the present study, subtyping of macrophages is limited since we did not perform specific antibodies to detect macrophage polarization. Nevertheless, our study confirmed that macrophages are an important component of the TME, constituting 25.4% of all GC-associated immune cells and affect clinical outcomes.

The density of B cells was generally associated with better survival although statistical significance was not reached in the present study. Similar with the current study, some researchers also demonstrated that B cells are associated with favorable prognosis in GC [23, 24], although others reported no specific association between B cells and prognosis [19]. B cells not only produce antibodies but also act as antigen presenting cells when properly activated [59]. These activated B cells can contribute to anti-tumor immune response by inducing both CD4 + and CD8 + T cells response [60, 61]. However, the prognostic role of B cells in GC is controversial to date and requires further validation.

Although the proportion of CD57 + NK cells was relatively low, higher tumoral CD57 + NK cells and tumoral CD57 + NKG2A + NK cell density was significantly associated with shorter OS and PFS. The prognostic implication of NK cells remains controversial across various types of cancer; some researchers have reported they were associated with good prognosis in lung cancer [62], and colorectal cancer [29], but others have demonstrated the opposite results in breast cancer [31], and glioblastoma [32]. In GC, a high density of NK cells was generally associated with improved clinical outcomes [30, 41, 63, 64]. A few studies have investigated the expression of tumor cell receptors which interact with NK cell surface receptors. Mimura et al. [42] reported that elevated expression of tumor cell MHC Class I chain-related A (MICA), MHC Class I chain-related B (MICB), and several UL-16-binding proteins (ULBPs), which are receptors for the NK cell-activating receptor, NKG2D, was associated with improved survival outcomes. On the other hand, Ishigami et al. [65] demonstrated that high expression of tumor cell HLA-E, which interacts with NKG2A, was associated with inferior outcomes. Our study revealed that not only CD57 + NKG2A + NK cells but also CD57 + NK cells in total were indicative of poor outcomes. Previous studies used single conventional IHC such as CD57, which renders it impossible to differentiate between CD57 + NK cells and CD57 + T cells may explain the discrepancy between the current study and the previous studies. As mentioned above, majority of CD57 + lymphocytes are CD8 + T cells, which might have affected the prognostic impact of CD57 marker, because higher CD8 + T cell infiltration is usually associated with better prognosis. By applying mIHC, we demonstrated that infiltration of CD57 + NK cells is, in fact, associated with inferior outcomes. Our results suggest that the cytotoxic function of CD57 + NK cells might be impaired in GC. Impaired NK cell function could be due to the expression of inhibitory receptors other than NKG2A, such as, killer cell immunoglobulin-like receptors (KIR), lymphocyte activation gene-3 (LAG-3), PD-1, or CTLA-4, which we did not include in the present study. In addition, researchers have demonstrated that transforming growth factor-beta (TGF-β) [66] or prostaglandin E2 (PGE2) [45] secreted by tumor-associated macrophages and tumor cells can inhibit NK cell cytotoxicity and proliferation in GC. Therefore, therapies that can restore the cytotoxic activity of NK cell, including the recently developed monoclonal NKG2A antibody, KIR antagonist, and inhibiting TGF-β signaling are expected to improve survival in GC patients.

Regarding molecular classification, EBV + GCs showed a higher density of T cells, which is in line with previous studies [35, 67]. The higher number of T cells in EBV + GCs is attributed to the relatively favorable clinical course in patients with EBV + GCs [68, 69]. Although the density of stromal and intratumoral CD57 + T cells differed according to the molecular subtypes, interpreting these results in this study is limited due to small sized sample. The significance of these differences with reference to clinical outcomes should be further validated in a large cohort.

Lastly, we demonstrated that HLA-E expression correlated with intratumoral T cells, B cells, and macrophages, but not CD57 + NK cells. Upon ligation with peptide-loaded HLA-E molecules on tumor cells, NKG2A induces the downregulation of NK cell function, thereby leading to immune escape. Ironically, HLA-E expression on tumor cells is induced by interferon-gamma (IFN-γ), which is produced by active immune cells [70]. A positive correlation between HLA-E expression in tumor cells and T cells [71] and NKG2A + immune cells [72] was reported, as well as a negative correlation with CD57 + immune cells [73]. Although our study failed to identify a direct correlation between HLA-E expression with CD57 + NK cells or NKG2A + cells, correlation with other immune cells suggests that HLA-E could be induced by active anti-tumor immune response possibly to bypass NKG2A + T and CD57 + NK cells.

## Conclusions

Our study has a limitation because this was a retrospective study and was composed of small sized cohort. However, we observed that intratumoral CD57 + NK cells and CD57 + NKG2A + NK cells were associated with poor outcomes. This suggests that NK cells play an important role in GC progression, although their density was the lowest among other immune cells. Therefore, targeting NK cell inhibitory receptors, such as NKG2A, may lead to successful GC treatment strategies.

## Supplementary Information

**Additional file 1: Table S1**. List of reagents and software used in the present study

**Additional file 2: Table S2**. The panel information and order of sequential IHC antibody.

**Additional file 3: Figure S1**. Representative figures of immune and tumor cells expressing CD3 (A), CD57 (B) and cytokeratin (C) (40×). Cells expressing each molecular marker were visualized by assigning colors, CD3 (D), CD57 (E), cytokeratin (F).

**Additional file 4: Table S3**. Immune cell density in spatial context.

**Additional file 5: Figure S2**. The proportion of each immune cells. T cells, macrophages, CD57+ NK cells, and B cells among CD16+ immune cells (A), and NKG2A+ immune cells (B).

**Additional file 6: Figure S3**. Comparison of immune cells according to EBV and microsatellite status. The number of B cells (A), macrophages (B), and CD57+ NK cells (C) did not differ significantly between EBV-negative gastric cancer and EBV-positive gastric cancer. Similarly, the number of T cells (D), B cells (E), macrophages (F), and CD57+ NK cells (G) did not differ significantly between microsatellite unstable gastric cancer and microsatellite stable gastric cancer.

## Data Availability

The authors declare that the data supporting the fndings of this study are available within the paper (and its supplementary information files). The additional datasets generated and/or analyzed during the current study are available from the corresponding authors upon reasonable request.

## Abbreviations

CTL: Cytotoxic T lymphocyte
CyTOF: Mass cytometry
EMT: Epithelial-mesenchymal transition
FDA: Food and drug administration
GC: Gastric cancer
ICI: Immune checkpoint inhibitor
IFN-γ: Interferone-gamma
IHC: Immunohistochemistry
ISH: In situ hybridization
KIR: Killer cell immunoglobulin-like receptors
LAG-3: Lymphocyte activation gene-3
MICA: MHC class I chain-related A
MICB: MHC class I chain-related B
mIHC: Multiplex immunohistochemistry
MSI: Microsatellite instability
MSI-H: MSI-high
MSI-L: MSI-low
MSS: Microsatellite stable
NK: Natural killer
OS: Overall survival
PCR: Polymerase chain reaction
PFS: Progression-free survival
PGE2: Prostaglandin E2
TGF-β: Transforming growth factor-beta
TMA: Tissue microarray
TME: Tumor immune microenvironment
ULBP: UL-16-binding protein
