# Supplementary material for: Prognostic significance of natural killer cell-associated markers in gastric cancer: quantitative analysis using multiplex immunohistochemistry
Source: J Transl Med. 2021 Dec 24;19:529. doi: 10.1186/s12967-021-03203-8 (PMC8710020; doi:10.1186/s12967-021-03203-8)
Supplement: Supplementary file 1 — Additional file 1: Table S1. List of reagents and software used in the present study [file 12967_2021_3203_MOESM1_ESM.docx]

| Table S1. List of reagents and software used in the present study. | |
| --- | --- |
| Category | Source |
| Reagent |  |
| Tris-EDTA buffer, pH 9.0 and citrate buffer, pH 6.0 | Zytomed Systems |
| Peroxidase-blocking solution | Dako |
| Washing buffer | Dako |
| Envision FLEX+ mouse linker/rabbit linker | Dako |
| Mouse anti-rabbit IgG/rabbit anti-mouse IgG | Invitrogen |
| Anti-rabbit/anti-mouse Envision+ System horseradish peroxidase | Dako |
| ImmPACT NovaRED | Vector Laboratories |
| 20% Sodium dodecyl sulfate | Promega |
| 0.5 M Tris-HCL pH 6.8 | Biosolutions |
| β-MercaptoethanolHarris hematoxylin | BioBasic Canada |
| Harris hematoxylin | Merk |
| Mayer's hematoxylin | Dako |
| Software |  |
| Aperio AT2 | Leica Biosystems |
| CellProfiler 3.1.8 | http://www.cellprofiler.org |
| R statistical package 3.5.3 | http://www.r-project.org |
