# Supplementary material for: Prognostic significance of natural killer cell-associated markers in gastric cancer: quantitative analysis using multiplex immunohistochemistry
Source: J Transl Med. 2021 Dec 24;19:529. doi: 10.1186/s12967-021-03203-8 (PMC8710020; doi:10.1186/s12967-021-03203-8)
Supplement: Supplementary file 2 — Additional file 2: Table S2. The panel information and order of sequential IHC antibody. [file 12967_2021_3203_MOESM2_ESM.docx]

| Table S2. The panel information and order of sequential IHC antibody. | | | | |
| --- | --- | --- | --- | --- |
| Order | Antibody | Clone | Dilution | Source |
| Round 1 | NKG2A | polycloncal | 1:100 | abcam |
| Round 2 | HLA E | polycloncal | 1:100 | abcam |
| Round 3 | CD20 | L26 | 1:300 | Dako |
| Round 4 | CD3 | polycloncal | 1:300 | Dako |
| Round 5 | CD57 | HNK-1/Leu-7 | 1:100 | abcam |
| Round 6 | CD45 | polycloncal | 1:600 | Novus |
| Round 7 | CD16 | SP175 | 1:100 | abcam |
| Round 8 | SMA | 1A4 | 1:1000 | ThermoFisher |
| Round 9 | CK | AE1/AE3 | 1:300 | Dako |
| Round 10 | ki67 | EPR3610 | 1:500 | abcam |
| Round 11 | CD68 | PG-M1 | 1:100 | Dako |
