# Supplementary figures and images for: Prognostic significance of natural killer cell-associated markers in gastric cancer: quantitative analysis using multiplex immunohistochemistry
Source: J Transl Med. 2021 Dec 24;19:529. doi: 10.1186/s12967-021-03203-8 (PMC8710020; doi:10.1186/s12967-021-03203-8)

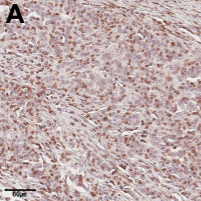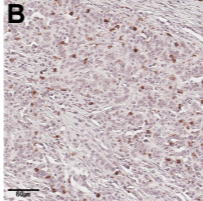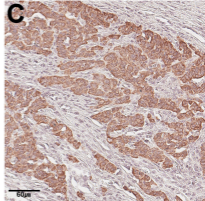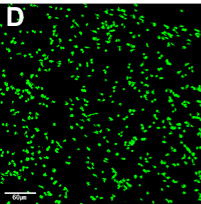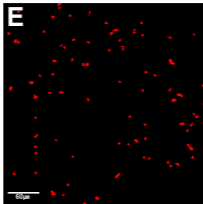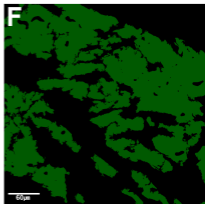

Supplement: Supplementary file 3 — Additional file 3: Figure S1. Representative figures of immune and tumor cells expressing CD3 (A), CD57 (B) and cytokeratin (C) (40×). Cells expressing each molecular marker were visualized by assigning colors, CD3 (D), CD57 (E), cytokeratin (F). [file 12967_2021_3203_MOESM3_ESM.pdf]
