# Supplementary material for: Prognostic significance of natural killer cell-associated markers in gastric cancer: quantitative analysis using multiplex immunohistochemistry
Source: J Transl Med. 2021 Dec 24;19:529. doi: 10.1186/s12967-021-03203-8 (PMC8710020; doi:10.1186/s12967-021-03203-8)
Supplement: Supplementary file 4 — Additional file 4: Table S3. Immune cell density in spatial context. [file 12967_2021_3203_MOESM4_ESM.docx]

| Table S3. Immune cell density in spatial context. | | | |
| --- | --- | --- | --- |
| Immune cell type | all (median, range)  (n/mm^2) | tumoral (median, range) (n/mm^2) | stromal (median, range) (n/mm^2) |
| T cell | 1281.2 (155.5-3180.1) | 1053.2 (54.5-2634.8) | 1281.5 (222.4-4066.5) |
| CD57+ T cell | 182.0 (17.1-1029.0) | 165.8 (6.7-982.5) | 201.8 (15.9-1383.1) |
| NKG2A+ T cell | 119.0 (2.0-637.7) | 89.2 (0.0-1291.8) | 119.6 (1.68-683.1) |
| B cell | 38.7 (0.0-963.4) | 12.7 (0.0-663.2) | 41.2 (0.0-992.5) |
| CD57+ NK cell | 44.6 (0.0-323.4) | 30.9 (0.0-791.4) | 40.9 (0.0-301.5) |
| CD57+CD16+ NK cell | 21.5 (0.0-171.5) | 15.1 (0.0-164.4) | 20.4 (0.0-217.3) |
| CD57+NKG2A+ NK cell | 3.24 (0.0-144.6) | 1.6 (0.0-619.5) | 3.4 (0.0-97.2) |
| Macrophage | 469.1 (40.3-1613.3) | 398.8 (21.1-1693.5) | 480.7 (53.0-1775.7) |
